# Beyond Pulmonary Vein Reconnection: Exploring the Dynamic Pathophysiology of Atrial Fibrillation Recurrence After Catheter Ablation

**DOI:** 10.3390/jcm14092919

**Published:** 2025-04-23

**Authors:** Panayotis K. Vlachakis, Panagiotis Theofilis, Anastasios Apostolos, Paschalis Karakasis, Nikolaos Ktenopoulos, Aristi Boulmpou, Maria Drakopoulou, Ioannis Leontsinis, Panagiotis Xydis, Athanasios Kordalis, Ioanna Koniari, Konstantinos A. Gatzoulis, Skevos Sideris, Costas Tsioufis

**Affiliations:** 1 1st Department of Cardiology, “Hippokration” General Hospital, National and Kapodistrian University of Athens, 11527 Athens, Greece; panos.theofilis@hotmail.com (P.T.); anastasisapostolos@gmail.com (A.A.); nikosktenop@gmail.com (N.K.); mdrakopoulou@hotmail.com (M.D.); giannisleontsinis@gmail.com (I.L.); panosxydis@yahoo.gr (P.X.); akordalis@gmail.com (A.K.); kgatzoul@med.uoa.gr (K.A.G.); ktsioufis@gmail.com (C.T.); 2 Second Department of Cardiology, Aristotle University of Thessaloniki, Hippokration General Hospital, 54124 Thessaloniki, Greece; pakar15@hotmail.com; 3 Third Cardiology Department, Hippokration University Hospital of Thessaloniki, 54642 Thessaloniki, Greece; aristibou@gmail.com; 4 Department of Cardiology, University Hospital of Patras, 26504 Patras, Greece; iokoniari@yahoo.gr; 5 State Department of Cardiology, “Hippokration” General Hospital of Athens, 11527 Athens, Greece; skevos1@otenet.gr

**Keywords:** atrial fibrillation, catheter ablation, atrial fibrillation recurrence, pathophysiology, cardiac electrophysiology

## Abstract

Atrial fibrillation (Afib) recurrence after catheter ablation (CA) remains a significant clinical challenge, driven by a complex and dynamic interplay of structural, electrical, and autonomic mechanisms. While pulmonary vein isolation (PVI) is the cornerstone of CA, recurrence rates remain substantial, highlighting the need to understand the evolving pathophysiology beyond PV reconnection. Post-ablation changes, including inflammation, edema, oxidative stress, and ischemia, create a transient proarrhythmic state that may contribute to early recurrence. Over time, atrial remodeling, fibrosis, and residual autonomic activity further sustain arrhythmogenicity. Additionally, epicardial adipose tissue promotes atrial myopathy, accelerating disease progression, particularly in patients with risk factors such as older age, female sex, obesity, hypertension, obstructive sleep apnea, and heart failure. The multifactorial nature of Afib recurrence underscores the limitations of a “one-size-fits-all” ablation strategy. Instead, a patient-specific approach integrating advanced mapping techniques, multimodal imaging, and computational modeling is essential. Artificial intelligence (AI) and digital twin models hold promise for predicting recurrence by simulating individualized disease progression and optimizing ablation strategies. However, challenges remain regarding the standardization and validation of these novel approaches. A deeper understanding of the dynamic interconnections between the mechanisms driving recurrence is crucial for improving long-term CA outcomes. This review explores the evolving nature of Afib recurrence, emphasizing the need for a precision medicine approach that accounts for the continuous interaction of pathophysiological processes in order to refine patient selection, ablation strategies, and post-procedural management.

## 1. Introduction

In 1909, Sir Thomas Lewis, a close associate of William Einthoven, was the first to record an electrocardiogram showing atrial fibrillation (Afib) in a patient with an irregular pulse rate [1]. Since then, Afib has become the most common cardiac arrhythmia encountered in daily clinical practice and remains a major global health concern, with data indicating a rising prevalence and incidence worldwide [2]. Its increase, especially in the aging population, contributes to significant morbidity and mortality—primarily through stroke and heart failure—highlighting the need for effective, long-term management strategies [3,4].

The knowledge of Afib pathophysiology, along with its detection and clinical management, has progressed considerably since Einthoven’s initial ECG recording. Pulmonary vein isolation (PVI) emerged as a cornerstone of catheter ablation (CA) for rhythm control following a pivotal work of Haïssaguerre et al. in the late 1990s [5]. Randomized controlled trials (RCTs) have since confirmed CA’s superiority over medical therapy in reducing Afib recurrence, establishing it as a preferred option for selected patients with paroxysmal or persistent forms [6,7,8]. Despite advancements in ablation technologies and procedural techniques, Afib recurrence remains a substantial challenge, with rates varying widely across studies due to differences in patient characteristics, follow-up duration, and definitions of success [9].

Traditionally, recurrence has been classified as early, late, or very late based on temporal patterns; however, this classification has become increasingly obsolete due to overlapping mechanisms and the complex interplay of structural, electrical, and autonomic factors that contribute to recurrence at different time points [10]. Instead, a more mechanistic approach to understanding Afib recurrence is warranted, focusing on the dynamic pathophysiological processes underlying atrial remodeling, autonomic dysfunction, and the persistence of arrhythmogenic substrates despite initial procedural success.

This review aims to shed light on the complex pathophysiological mechanisms contributing to Afib recurrence after CA, emphasizing their dynamic interplay to better understand and optimize patient outcomes.

## 2. Pathophysiology of Afib Recurrence After Catheter Ablation

The mechanisms underlying Afib recurrence after CA are dynamic and multidimensional, involving a complex interplay of post-ablation changes, pre-existing atrial substrate characteristics, and patient-specific factors.

### 2.1. Pre-Ablation Atrial Substrate and Patients’ Factors

The pre-ablation atrial substrate varies by Afib type and significantly influences recurrence risk post-CA. Long-standing persistent Afib is linked to more extensive remodeling compared with persistent or paroxysmal forms [11]. In line with the concept that “Afib begets Afib”, prolonged Afib duration in persistent cases drives complex pathophysiological pathways, leading to atrial remodeling, fibrosis, and conduction abnormalities [12]. This creates a proarrhythmic milieu that increases the risk of recurrence post-CA [13]. These structural and electrical alterations are less severe in paroxysmal Afib, correlating with lower recurrence rates [14].

Regarding patient factor, age significantly influences Afib recurrence risk post-CA, with individuals over 65-years exhibiting extensive atrial fibrosis and structural remodeling that heighten arrhythmogenic potential [15]. A meta-analysis of 108,419 patients indicated a 24–64% increased recurrence risk in those ≥75 years, particularly with persistent Afib, while Boehmer et al.’s study of 873 patients post-cryoballoon ablation confirmed a linear rise with age due to progressive substrate deterioration [15,16]. Sex differences also play a critical role, with women exhibiting a 76% greater recurrence risk than men, possibly linked to advanced fibrotic substrates and hormonal influences [17,18]. This disparity may explain fewer women undergoing CA, reflecting delayed access to invasive options alongside smaller left atrium (LA) size, lower LA voltage, and reduced pericardial fat volume compared with men [19]. Additionally, women show a higher prevalence of non-pulmonary vein (non-PV) foci—key recurrence drivers—and more low-voltage areas indicative of fibrotic remodeling, further elevating their post-CA risk [20].

Various comorbidities linked to Afib incidence have been explored as potential contributors to recurrence post-CA, however, their persistent influence on creating an arrhythmogenic substrate lacks firm confirmation in current research [21]. Conditions like diabetes mellitus, chronic kidney disease, and coronary artery disease are sporadically associated with heightened recurrence risk, but robust evidence remains elusive. Among these, hypertension (HTN) distinctly alters the pre-ablation atrial substrate, increasing the recurrence likelihood through unique pathophysiological pathways [22]. Sustained HTN elevates atrial pressure overload and activates the renin-angiotensin-aldosterone system (RAAS), driving oxidative stress and inflammation that disrupt atrial electrical stability [23]. This proarrhythmic milieu, compounded by left ventricular hypertrophy and reduced atrial compliance, fosters ectopic activity and challenges PVI success [24,25]. Severe HTN intensifies these effects, raising procedural complexity and recurrence rates, often requiring repeat interventions [24]. Effective HTN management may counteract these substrate changes, enhancing CA outcomes and underscoring its pivotal role in recurrence dynamics [26].

Elevated body mass index (BMI) is strongly associated with persistent Afib and an increased risk of recurrence after CA [27,28]. Obesity drives atrial structural and electrical remodeling, including increased LA pressure, interstitial fibrosis, and reduced conduction velocity, as observed in preclinical models [29]. These changes, accompanied by elevated transforming growth factor-beta 1 (TGF-β1) and diminished connexin-43, create a proarrhythmic substrate that undermines ablation efficacy [29]. Epicardial adipose tissue (EAT), more strongly linked to Afib than overall adiposity, further exacerbates this through inflammation and fatty infiltration [29]. Clinical studies, such as ARREST-AF and LEGACY, demonstrated that weight loss (e.g., >10%) reduces the recurrence risk by mitigating these substrate alterations, highlighting their reversibility [30,31]. Finally, alcohol consumption exacerbates Afib recurrence post-CA by enlarging the LA diameter and increasing low-voltage zones beyond the pulmonary veins, reflecting a fibrotic atrial myopathy that resists pulmonary vein isolation and promotes new arrhythmogenic foci [32,33].

### 2.2. Ablation-Induced Myocardial Injury, Inflammation, and Atrial Fibrosis

Early recurrence of Afib, occurring within the first 3 months post-ablation, is influenced by several mechanisms related to the healing process, inflammation, and tissue remodeling in the atria [34]. The rate of early Afib recurrence within the 3-month period after PVI varies between 9% and 65% [35,36]. This period, often referred to as the “blanking” period, involves various temporary proarrhythmic processes that are not necessarily predictive of long-term outcomes but significantly impact early Afib recurrence [37]. Recently, the EHRA expert consensus document shortened the traditional 3-month blanking period to 8-weeks to improve the classification of patients with early recurrences. This change is supported by studies showing that the risk of long-term Afib relapse increases as recurrences occur later within the first 3 months [10].

From a pathophysiological perspective, ablation induces localized necrosis due to energy delivery, causing myocardial injury and releasing troponin, which peaks on the first day post-ablation [34]. Elevated troponin levels immediately after CA have been associated with early Afib recurrence within the first few days of the blanking period [38]. However, this biomarker does not appear to reliably predict late recurrence [39]. Interestingly, Yoshida et al. observed that in patients with persistent Afib and an enlarged left atrium (LA), those who experienced Afib recurrence at 2 months had lower PVI-induced troponin elevations than those without recurrence [40]. This may suggest that in these patients, ablation caused less myocardial loss, preserving more electrical mass and potentially contributing to a higher probability of recurrence.

Furthermore, ablation-induced atrial ischemia, caused by direct arterial trauma, thromboembolism, collagen shrinkage, and coronary spasm, can lead to early Afib recurrence [41]. Histological observations confirm hemorrhage and thrombosis near recent radiofrequency lesions, while ischemia is known to trigger proarrhythmic changes in the atria, as evidenced by the association between acute atrial myocardial infarction and Afib onset in humans as well as increased Afib inducibility in ischemic canine models [42,43,44]. Electrically active ablated tissue remains coupled to viable tissue, causing conduction slowing, action potential changes, and partial uncoupling, all of which promote arrhythmogenesis [45,46]. Heat-induced arterial narrowing develops rapidly post-ablation, potentially leading to chronic ischemia and a sustained proarrhythmic environment, supported by studies showing that atrial coronary artery disease predicts Afib after ventricular myocardial infarction [47,48]. Complete arterial occlusion can also cause myocardial necrosis extending beyond the ablation site, promoting fibrosis and the formation of re-entrant circuits that further sustain Afib [45,49].

The extent and nature of these tissue responses may vary depending on the energy source used during ablation, contributing to differences in healing and proarrhythmic potential. Importantly, different ablation modalities induce varying levels of tissue injury. Radiofrequency ablation causes volumetric heating and indiscriminate protein denaturation, often affecting the surrounding vasculature and delaying healing. Cryoablation leads to membrane damage via freeze–thaw mechanisms and microvascular clogging, while pulsed field ablation (PFA), through non-thermal electroporation, selectively disrupts cell membranes with minimal extracellular or vascular damage, enabling faster healing [50].

*Reactive oxygen species* (ROS) generated from ischemic injury post-ablation contribute to oxidative stress, a significant driver of arrhythmogenic activity [51]. Studies have shown that elevated ROS markers such as myeloperoxidase are associated with an increased likelihood of Afib recurrence within the first week post-ablation [52]. This stress response may be transient, as antioxidant markers generally decline after the blanking period [51]. Moreover, the formation of edema in ablated myocardial tissue disrupts electrical conduction and may generate proarrhythmic activity. Edema appears in the atrial tissue immediately after ablation and typically resolves within 1 to 3 months, making it a transient phenomenon [53]. This swelling may contribute to early recurrence by altering electrical conduction and transmembrane ionic currents as well as increasing the spacing around gap junctions, which can slow conduction [54,55]. Additionally, cell edema can activate specific ion channels, such as swelling-activated chloride channels, which may depolarize the resting membrane and influence arrhythmogenicity [56].

Ablation triggers both local and systemic inflammation, marked by increased C-reactive protein (CRP) levels peaking within days of the procedure [38]. Inflammation in the atrial tissue can disrupt conduction properties and promote Afib, and elevated CRP levels have been linked to higher rates of early recurrence [57]. Anti-inflammatory therapies, such as steroids, have shown some efficacy in reducing early Afib episodes, supporting the role of inflammation in the pathophysiology of early recurrence [58]. Enhanced “NACHT, LRR, and PYD Domains-containing Protein 3” (NLRP3)-inflammasome activity in atrial tissue, observed in patients with prior Afib or susceptibility to postoperative Afib, promotes recurrence post-CA by driving aberrant RyR2-mediated calcium release and shortening atrial effective refractory periods (AERPs), thus heightening Afib inducibility [59,60,61,62]. This proarrhythmic substrate is further exacerbated by obesity- or chronic kidney disease-related inflammasome activation, which fosters atrial remodeling and fibrosis, creating a condition conductive to sustained Afib despite ablation efforts [63]. Disruption to autonomic nerve fibers near the pulmonary veins (PVs) can alter parasympathetic and sympathetic tone, leading to increased susceptibility to Afib [64]. Both sympathetic activation and autonomic reinnervation can play a role in early recurrence by promoting proarrhythmic electrical changes in atrial tissue [65].

The necrotic tissue created by ablation undergoes a repair process, transforming into a mature collagenous scar within the blanking period. This scar formation progresses through stages: initial necrosis, collagen crosslinking, and finally, the development of a dense collagen matrix by weeks 6 to 8 post-ablation [66,67]. Scar maturation stabilizes by three months, aligning with a reduced likelihood of Afib recurrence; patients with denser scar tissue generally show lower rates of late recurrence compared with those with less extensive scarring [68]. Finally, a complicating factor in understanding the pathophysiological mechanisms during the blanking period is the routine use of AADs, typically discontinued afterward. While AADs are often prescribed to support sinus rhythm (SR) and reduce early recurrence within this period, evidence suggests that they do not enhance long-term outcomes and may even have proarrhythmic effects that mask or delay a true (anti)arrhythmic response post-ablation [69,70,71].

### 2.3. Pulmonary Vein Reconnection

PV reconnection is a primary pathophysiological mechanism underlying Afib recurrence. Structural and electrophysiological remodeling processes contribute to the reconnection of previously isolated PVs, facilitating the recurrence of Afib. Several factors, including older age, male sex, larger LA size, and early recurrence, have been linked to an increased risk of late recurrence, reflecting the progressive nature of atrial remodeling [72,73,74,75].

Fibrotic remodeling, heterogeneity in conduction, and altered electrophysiological properties of the PV–LA junction are central to PV reconnection. In repeat electrophysiology procedures, at least one PV reconnection has been detected in 98% of patients with clinically evident late Afib recurrence, reinforcing its critical role in the long-term failure of PVI [76]. Moreover, in a study of 2886 patients who initially underwent PVI, 6% required three or more ablations, with 92% demonstrating PV reconnection. Among these, 41% exhibited reconnection in all four PVs, while only 8% maintained complete PV isolation, underscoring the progressive nature of electrical remodeling in Afib [77]. The interplay between fibrosis formation and incomplete lesion maturation appears to be a key determinant of PV reconnection. Persistent conduction gaps in ablation lesions, driven by heterogeneous tissue recovery, may create a substrate for reentrant circuits, increasing the likelihood of recurrence. Studies have demonstrated that re-isolating these reconnected PVs leads to a significant suppression of Afib recurrence, suggesting that PV reconnection remains the dominant pathophysiological driver of late recurrence [78].

Advanced imaging modalities, particularly late gadolinium enhancement (LGE) MRI, have emerged as potential tools for assessing lesion integrity and fibrosis formation. The extent of post-ablation fibrosis correlates with long-term outcomes, and complete LGE lesions encircling the PVs have a negative predictive value exceeding 90% for PV reconnection during invasive testing [79]. The optimal window for detecting ablation-induced fibrosis via LGE-MRI is approximately three months post-ablation [80]. However, while some data suggest that pre-procedural LGE-MRI for substrate characterization may improve repeat ablation outcomes, the results remain inconclusive, necessitating further investigation through larger, randomized trials [81].

Although electrophysiological mapping remains the gold standard for detecting PV reconnection, noninvasive techniques such as premature atrial complex analysis, electrocardiographic imaging, and artificial intelligence-driven ambulatory monitoring are being investigated for their potential to identify reconnection mechanisms [82,83]. These approaches may offer valuable insights into the evolving atrial substrate, providing a more individualized assessment of recurrence risk.

### 2.4. Non-Pulmonary Vein Triggers

Non-pulmonary vein (non-PV) triggers play a significant role in the re-initiation of Afib following CA [84]. Data from studies show that the estimated prevalence of non-PV triggers ranges from 3% to 47% [85,86]. These non-PV triggers are particularly prevalent in patients with persistent Afib and those with risk factors such as advanced age, female sex, sleep apnea, obesity, structural remodeling of the atria, HF, cardiomyopathy, or valvular heart disease [87]. These triggers arise from embryologically distinct atrial regions with unique electrophysiological properties that facilitate arrhythmogenesis [84].

The LA posterior wall, embryologically derived from PV tissue, exhibits shorter action potential durations, refractory periods, and pacemaker-like activity [88]. It also harbors ganglionated plexi (GP) storing acetylcholine, which increases potassium currents, promoting reentry circuits [89,90]. Additionally, heterogeneous fiber orientation and fibrous-fatty infiltration within the posterior wall contribute to conduction block and anisotropic propagation, reinforcing Afib maintenance [91]. Its isolation has thus emerged as a key strategy in Afib rhythm control.

The superior vena cava (SVC) contains atrial myocardial sleeves with automaticity, serving as an Afib initiator [92]. Atrial ectopy from longer muscular sleeves (>30 mm) and high-amplitude potentials (>1 mV) increases Afib risk, with SVC-related triggers identified in 5.2% of Afib patients, of whom 78.4% exhibited SVC-initiated Afib [93]. While primarily an initiator, the SVC may also perpetuate Afib, though its role diminishes in long-standing Afib, where other non-PV triggers take over [94].

The coronary sinus (CS) and vein of Marshall (VOM) contribute to Afib perpetuation through discontinuous myocardial bundles, forming slow conduction pathways that promote reentry and ectopic activity [84]. Although one earlier study suggested that “complete” CS isolation might be more effective than focal ablation in eliminating CS-related triggers [95], this finding has not been consistently supported by more recent research. Therefore, a more targeted ablation approach within the CS may be considered, especially in the context of identifying extra-PV foci. Notably, the presence of a persistent left SVC has been associated with arrhythmogenic foci in the CS region, as reported in several case reports and case series [96,97,98]. The VOM, a remnant of the left superior vena cava, contains autonomic nerve fibers and electrical connections to the PVs, LA, and CS, explaining why up to 30.2% of post-ablation left atrial tachyarrhythmias are Marshall-bundle related [99].

Although not widely recognized as a major trigger site, the interatrial septum (IAS) shows low-voltage areas linked to Afib in patients with sleep apnea and paroxysmal Afib [100]. Its preferential conduction pathways and vascularized adipose content create substrates for reentry, and targeted ablation in this region has been associated with reduced Afib recurrence (64% vs. 84% in PVI-only patients, *p* = 0.003) [101].

The left atrial appendage (LAA) has emerged as a critical non-PV trigger site, particularly in non-paroxysmal Afib [102]. It contains pacemaker-like cells, and its fibrosis, dilation, and muscle atrophy in chronic Afib facilitate localized reentry circuits [103]. Anatomical connections to Bachmann’s bundle and the CS enable rapid Afib propagation, making LAA ablation a key consideration in refractory Afib [104].

The crista terminalis (CT) in the right atrium harbors sinoatrial-like pacemaker extensions and anisotropic conduction, making it an Afib substrate [105]. Ectopic beats from the CT can initiate and perpetuate Afib, with the conduction block promoting multiple wavefront reentry circuits [106]. Ablation of CT-related tachycardia has been shown to significantly reduce Afib recurrence over long-term follow-up [84].

In summary, these extrapulmonary trigger sites have increasingly been integrated into tailored ablation strategies for patients with recurrent or non-paroxysmal Afib. Posterior wall isolation, particularly with a wide antral approach, has demonstrated superior long-term efficacy over ostial PVI in meta-analyses and select series [107,108,109,110]. Non-PV triggers in regions such as the SVC, LAA, and CS are typically addressed through complete isolation, which has been associated with improved outcomes [111]. Empirical LAA isolation, as shown in the BELIEF trial, enhanced arrhythmia-free survival in long-standing persistent Afib patients, while ethanol infusion of the VOM has emerged as an effective adjunct in achieving mitral isthmus block and reducing recurrence, though with technical limitations [112,113,114]. These findings support the expanding role of non-PV sites as critical targets in advanced ablation strategies, especially in patients with complex substrates or prior ablation failure.

### 2.5. Autonomic Nervous System Modulation

The autonomic nervous system (ANS), particularly GP, plays a crucial role in Afib initiation, maintenance, and recurrence. GPs, located near PV ostia and other cardiac sites, regulate electrical activity by integrating sympathetic and parasympathetic inputs [115]. Increased vagal tone from GP stimulation shortens the atrial effective refractory period (AERP), promoting Afib triggers and reentry circuits [116]. Ablation of GPs aims to disrupt these autonomic inputs, but challenges remain regarding precise GP localization, depth, and complete ablation. Anatomical variability, difficulty targeting deeper plexi, and the potential for GP regeneration contribute to procedural limitations [117]. Additionally, thermal ablation methods pose risks such as cardiac tamponade, PV stenosis, esophageal fistula, and thrombus formation [118]. While some studies report reduced Afib burden with GP ablation alone, long-term efficacy remains uncertain, particularly in advanced Afib, where structural remodeling diminishes the impact of ANS modulation [119]. A large randomized trial during thoracoscopic surgery found no significant benefit of GP ablation in such patients [120].

Given the limitations of GP ablation alone, its combination with PVI has been explored to improve outcomes. PVI addresses focal triggers, while GP ablation targets the autonomic substrate sustaining Afib. Short-term follow-ups show success rates improved by 20–28% compared with PVI alone, but the long-term benefits (beyond two years) are more modest, with success rates increasing only by 2.5–8% [120,121]. Variability in study protocols, unclear GP targeting, and potential overlap between PVI lesions and GP sites complicate data interpretation. Heart rate variability (HRV) has emerged as a predictor of ablation success, with increased post-procedural heart rates correlating with lower Afib recurrence [122]. Despite its importance, AERP measurements remain underutilized due to technical challenges, though studies suggest a link between AERP shortening and AF persistence.

Similarly, renal denervation (RDN) plays a key role in autonomic modulation and Afib recurrence by disrupting renal sympathetic nerves, reducing both sympathetic input to the central nervous system and RAAS activation [123]. The ERADICATE-AF trial showed that adding RDN to PVI in hypertensive patients significantly improved AF freedom at 12 months (72.1% vs. 56.5%, *p* = 0.006) and lowered blood pressure. While the exact mechanism remains unclear—whether due to blood pressure reduction or direct sympathetic denervation—meta-analyses have confirmed its benefit (OR 0.63, 95% CI [0.50–0.80], *p* < 0.001) [123]. Despite the lack of a sham control, RDN may emerge as a viable strategy for hypertensive patients undergoing Afib ablation.

### 2.6. Epicardial Adipose Tissue (EAT)

EAT is distinguished by its direct adjacency to the heart, lacking a separating muscular fascia, and by its unique transcriptome and secretome, which set it apart from other depots [124]. This anatomical and biochemical proximity facilitates a bidirectional interaction with the underlying myocardium via a shared microcirculation, positioning EAT as a significant contributor to Afib recurrence following CA. Studies have identified EAT as an independent predictor of Afib development and post-ablation recurrence, with greater thickness or volume observed in patients with chronic or persistent Afib compared with those with paroxysmal Afib, irrespective of obesity or conventional risk factors [125]. The peri-atrial EAT, in particular, exhibits distinct properties that amplify its arrhythmogenic potential, influencing the atrial substrate in ways that undermine ablation efficacy [126].

The pathophysiological role of EAT in Afib recurrence post-CA is multifaceted, encompassing inflammation, fibrosis, fatty infiltration, and neural modulation. Peri-atrial EAT is enriched with genes encoding pro-inflammatory adipokines, such as interleukins and tumor necrosis factor (TNF), and profibrotic factors including matrix metalloproteinases, connective tissue growth factor, transforming growth factor-beta (TGF-β1, TGF-β2), and activin A [127,128]. These bioactive molecules can diffuse into the adjacent atrial myocardium, promoting inflammatory milieu and fibrotic remodeling that persist beyond the ablation procedure. Evidence from patients with Afib shows that EAT-derived extracellular vesicles contain profibrotic cytokines and microRNAs, further driving atrial fibrosis [129]. This fibrotic deposition disrupts electrical conduction by slowing propagation and creating zones of conduction block, fostering re-entrant circuits that sustain AF despite initial ablation success [130].

Additionally, EAT serves as a physiological reservoir of free fatty acids for the atrium, a function that turns pathological under stress conditions [124]. Excessive free fatty acid infiltration from EAT into the atrial myocardium can physically separate cardiomyocytes, impairing side-to-side cell connections and causing myocardial disorganization [131,132]. This disruption leads to conduction delays or blocks, enhancing the substrate for re-entry and increasing the likelihood of Afib recurrence post-CA. Neural modulation by EAT further exacerbates this risk, as activation of its intrinsic ganglia shortens the action potential duration and amplifies the calcium transient amplitude, facilitating both the initiation and perpetuation of Afib [132]. These electrophysiological alterations may persist or emerge after ablation, particularly in regions not targeted by PVI such as the peri-atrial areas rich in EAT.

The dynamic interaction between EAT and the post-ablation atrial environment remains a critical but underexplored factor in recurrence. Ablation-induced injury, such as inflammation and scar formation, may amplify EAT’s arrhythmogenic effects by enhancing its secretome activity or fatty infiltration, but the extent of this interplay is not fully delineated. The accumulation of peri-atrial EAT, often more pronounced in persistent Afib, likely contributes to the higher recurrence rates observed in these patients, as it perpetuates a proarrhythmic substrate resistant to standard ablation strategies [125]. This suggests that EAT’s role extends beyond a static risk factor, acting as a modifiable determinant of long-term outcomes post-CA. While EAT can be quantified using echocardiography or cardiac computed tomography [124], its specific contribution to recurrence mechanisms warrants further investigation to refine the ablation approaches and adjunctive therapies targeting this tissue.

**Figure 1:**
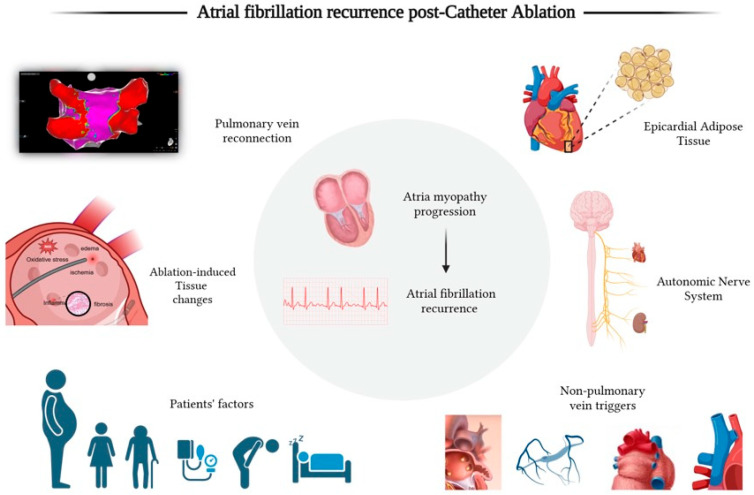
This figure illustrates the pathophysiological mechanisms involved in the recurrence of atrial fibrillation post-ablation. In the center, atrial myopathy progression leads to recurrence, with mechanisms contributing to this process shown outside the circle. These include pulmonary vein reconnection, post-ablation pathophysiological changes (such as inflammation, edema, oxidative stress, and ischemia), and non-pulmonary ectopic triggers (from left to right: left atrial appendage, vein of Marshall, posterior atrial wall, and superior vena cava). The figure also highlights the role of epicardial adipose tissue and the autonomic nervous system in sustaining arrhythmogenicity. Additionally, patient factors (older age, female sex, obesity, hypertension, obstructive sleep apnea, and heart failure) accelerate atrial myopathy progression through structural and electrical remodeling. Created with BioRender.com.

Figure 1 summarizes and provides an integrated overview of the multifactorial contributors to AFib recurrence, emphasizing the complex interplay between anatomical, physiological, and patient-specific factors.

## 3. Current State and Future Directions

CA for Afib has undergone notable improvements in safety, effectiveness, and the technological approaches during the procedure. The Heart Rhythm Society (HRS) defines successful CA as the absence of symptomatic or asymptomatic Afib, atrial tachycardia, or atrial flutter episodes lasting 30 s or more post-ablation [9]. The 2024 consensus from the European Heart Rhythm Association (EHRA)/Heart Rhythm Society (HRS)/Asia Pacific Heart Rhythm Society (APHRS)/Latin American Heart Rhythm Society (LAHRS) recognizes that this 30-s criterion may not fully capture the symptom intensity or cardiovascular impacts but retains it as a benchmark for consistency with prior studies [10]. Given that episodes exceeding one hour are deemed more clinically relevant, the latest EHRA/HRS/APHRS/LAHRS document advises a detailed outcome analysis—including Afib burden, results with or without antiarrhythmic drugs (AADs), and single versus multiple procedure metrics—for a thorough assessment [10]. One-year success is characterized by no arrhythmic events without AADs from the end of the blanking period (typically 8 weeks post-ablation) to 12 months [9]. Long-term success extends this arrhythmia-free period off AADs from the blanking phase to at least 36 months [9].

A critical issue in evaluating the success of Afib ablation is the need for standardized and clinically meaningful outcome measures. The current definition of success, which is based on the absence of 30-s atrial arrhythmia episodes, has limited clinical relevance. While Afib burden is a more meaningful metric, its accurate assessment requires continuous monitoring via implantable or wearable devices, which are rapidly evolving [133,134]. Future research should focus on developing universal efficacy endpoints that reflect patient-centered outcomes such as quality of life, exercise capacity, and reductions in major adverse cardiovascular events including stroke and mortality.

**Figure 2:**
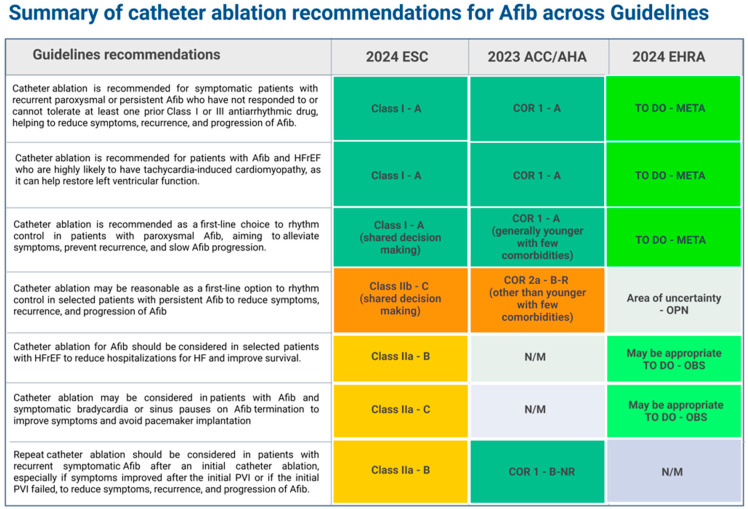
Summary of the key recommendations (class and level of evidence) for catheter ablation in atrial fibrillation based on the 2024 ESC Guidelines, 2023 ACC/AHA Guidelines, and 2024 EHRA/HRS/APHRS/LAHRS Consensus Statement. Recommendations included in this table reflect consensus, indicated by agreement from more than two of the three guideline documents. Abbreviations: *Afib*, atrial fibrillation*; ACC*, American College of Cardiology; *AHA*, American Heart Association; *COR*, class of recommendation; *EHRA*, European Heart Rhythm Association; *ESC*, European Society of Cardiology; *HFrEF*, heart failure with reduced ejection fraction; *OBS*, observational studies; *OPN*, randomized, non-randomized, observational or registry studies with limitations of design or execution, case series; *META*, meta-analysis of high-quality randomized clinical trials; *NR*, non-randomized; *N/M*, not mentioned; *PVI*, pulmonary vein isolation; *R*, randomized; *STEMI*, ST-segment elevation myocardial infarction.

Both the European Society of Cardiology (ESC) and American Heart Association (AHA) guidelines recommend (Class I) CA for patients with paroxysmal or persistent Afib who are resistant or intolerant to AADs, as CA can reduce the symptoms, recurrence, and progression of Afib. Additionally, CA is recommended for patients with a high probability of tachycardia-induced cardiomyopathy to reverse the underlying left ventricular systolic dysfunction. Furthermore, repeat CA should be considered in patients with recurrent symptomatic Afib following initial ablation, particularly if there was symptomatic improvement after the first PVI or in cases of PVI failure to further reduce symptom burden, recurrence, and disease progression (Figure 2) [135,136]. Nevertheless, Afib recurrence following ablation represents a substantial clinical challenge, affecting patient quality of life and long-term prognosis. Research indicates that recurrence rates range from 20% to over 50% within five years post-procedure, heavily dependent on the monitoring protocol’s type and thoroughness [37]. The electrophysiology community remains committed to deciphering the complex “enigma” of Afib recurrence by exploring the intricate and interwoven pathophysiological mechanisms at play, particularly the dynamic interactions contributing to atrial cardiomyopathy both before and after ablation. These efforts aim to refine ablation strategies and optimize long-term patient outcomes.

The pathophysiology of Afib recurrence is a dynamic and evolving process, influenced by patient-specific factors such as atrial remodeling, fibrosis, inflammation, and autonomic modulation. This complexity underscores the need to move beyond a “one-size-fits-all” ablation strategy. Electrophysiologists must adopt a more personalized, mechanism-driven strategy to Afib management, which includes integrating advanced mapping technologies, individualized ablation targets, and addressing modifiable patient risk factors both before and after the procedure. Managing obesity, treating underlying sleep apnea, and optimizing heart failure therapy are essential steps that should be considered to improve procedural success [137,138]. Additionally, pharmacological interventions, such as SGLT-2 inhibitors and psychological support for depression may contribute to reducing the recurrence rates [139]. By addressing these factors comprehensively, the likelihood of successful long-term outcomes can be significantly enhanced, minimizing the risk of Afib recurrence (Table 1).

Despite decades of research, the mechanisms driving Afib are not fully understood, hindering the development of optimal treatment strategies. A debate persists between the multiple wavelet hypothesis and focal sources as the primary drivers of Afib. Early mapping technologies have provided limited clinical improvements, but emerging tools show promise [140]. Persistent Afib is now recognized as a heterogeneous condition involving multiple, patient-specific mechanisms [11]. Future research should focus on advanced mapping techniques that allow for personalized ablation strategies, improving both the short- and long-term outcomes while minimizing unnecessary tissue destruction.

PVI remains the cornerstone of ablation for paroxysmal Afib, but pulmonary vein reconnection continues to be a major challenge contributing to arrhythmia recurrence. Despite decades of refinement, no adjunctive strategy has consistently outperformed PVI alone for persistent Afib, and outcomes remain suboptimal. Advances in computational modeling and machine learning may help identify optimal ablation targets, enabling more precise and individualized treatment approaches [141]. However, rigorous, multicenter studies are necessary to validate these innovations before widespread clinical implementation.

Artificial intelligence (AI) has emerged as a promising tool for predicting Afib recurrence following CA [142]. While key predictors such as LA global longitudinal strain (LA GLS) are well-documented, underutilized metrics—including regional LA strain, strain rate, and time-to-peak—may enhance prognostic accuracy [143,144]. AI’s ability to analyze time-series data, including complete strain curves, could further improve predictive accuracy beyond conventional peak strain measurements [144]. By integrating diverse data sources such as clinical metrics, cardiac imaging, and electrophysiological data, AI-driven machine learning and deep learning techniques can uncover complex patterns and relationships that were previously undetectable, offering novel insights into Afib pathophysiology and treatment response [145]. Additionally, radiomics features from cardiac CT and MRI-based simulations show promise but require validation in larger patient cohorts [146]. However, despite these advancements, concerns remain regarding the “black-box” nature of AI models and the potential biases introduced by unrepresentative training datasets, highlighting the necessity for cautious, transparent, and equitable implementation to ensure that AI-derived insights translate into meaningful patient benefits [147].

An emerging concept in this domain is the use of digital twins—virtual patient-specific models that integrate multimodal data, including imaging, electrophysiology, and genetic markers, to simulate disease progression and predict treatment outcomes [148]. These AI-driven simulations could revolutionize Afib management by allowing electrophysiologists to test various ablation strategies in silico before applying them in real patients, potentially improving both the safety and efficacy. However, the successful implementation of digital twins in clinical practice will require robust validation studies and seamless integration with the existing workflows.

## 4. Conclusions

Afib recurrence after CA is a complex and dynamic process driven by the intricate interplay of structural, electrical, and autonomic mechanisms, which continuously evolve over time. The multifactorial nature of these mechanisms underscores the limitations of a uniform ablation strategy and highlights the necessity of an individualized, mechanism-guided approach. PV reconnection remains a dominant driver of recurrence, but the contribution of non-PV triggers, atrial fibrosis, inflammation, and autonomic modulation must be acknowledged for a comprehensive treatment strategy. The integration of advanced mapping technologies, multimodal imaging, and AI holds promise for refining ablation targets and enhancing long-term success. AI-driven digital twin models, capable of simulating patient-specific disease progression and treatment responses, could revolutionize Afib management by allowing the pre-procedural optimization of ablation strategies. However, challenges related to standardization, clinical validation, and real-world implementation persist. Moving forward, a deeper understanding of the interwoven mechanisms of recurrence, coupled with precision medicine approaches, will be essential in addressing this ongoing clinical challenge and improving the long-term patient outcomes.

## Figures and Tables

**Table 1:** Emerging strategies to improve the atrial fibrillation ablation outcomes.

Strategy	Mechanism	Potential Benefit	Current Challenges
**Advanced Mapping Technologies**	AI-driven electrogram analysis, charge density mapping, digital twins	Improves identification of arrhythmogenic regions and ablation precision	Standardization and validation in large-scale clinical trials needed. Temporal resolution with multidiscipline catheters is a current limitation. It may also be worth splitting this into contact and non-contact mapping approaches
**Pulsed Field Ablation (PFA)**	Non-thermal ablation minimizing collateral damage	Reduced risk of esophageal, phrenic nerve, and pulmonary vein injury	Long-term efficacy and optimal lesion durability require further study
**Targeting Non-PV Triggers**	Ablation of LAA, CS, SVC, and posterior wall	Addresses drivers beyond PV reconnection to reduce recurrence	Complex mapping and risk of atrial tachycardia post-isolation. Heterogeneity in response to empirical approaches indicates these are not triggers in all patients. There is a need to develop individualized pathophysiology-based treatment approaches
**Hybrid Ablation Approaches**	Combination of endocardial and epicardial ablation	Enhances lesion durability, especially in persistent AF	Higher procedural risk and need for standardized protocols
**Autonomic Modulation (GP Ablation, RDN)**	Alters vagal and sympathetic tone to reduce Afib triggers	Potential adjunct to PVI in selected patients	Variable success rates and risk of autonomic reinnervation
**Risk Factor Optimization**	Weight loss, OSA treatment, blood pressure control	Modifies atrial substrate to improve long-term outcomes	Patient adherence and long-term sustainability of interventions
**AI & Computational Modeling**	Machine learning prediction models and digital twins	Enables personalized ablation strategies and risk assessment	Requires integration into clinical workflows and validation across diverse populations

**Abbreviations:** *AI*, artificial intelligence; *Afib*, atrial fibrillation; *CS*, coronary sinus; *GP*, ganglionated plexi; *LAA*, left atrial appendage; *OSA*, obstructive sleep apnea; *PFA*, pulsed field ablation; *PV*, pulmonary vein; *PVI*, pulmonary vein isolation; *RDN*, renal denervation; *SVC*, superior vena cava.

## Data Availability

Not applicable.
